# Association between cigarette smoking and the vaginal microbiota: a pilot study

**DOI:** 10.1186/1471-2334-14-471

**Published:** 2014-08-28

**Authors:** Rebecca M Brotman, Xin He, Pawel Gajer, Doug Fadrosh, Eva Sharma, Emmanuel F Mongodin, Jacques Ravel, Elbert D Glover, Jessica M Rath

**Affiliations:** Institute for Genome Sciences, University of Maryland School of Medicine, Baltimore, MD USA; Department of Epidemiology and Public Health, University of Maryland School of Medicine, 801 West Baltimore Street, Room Number 633, Baltimore, MD 21201 USA; Department of Epidemiology and Biostatistics, University of Maryland School of Public Health, College Park, MD USA; Department of Behavioral and Community Health, University of Maryland School of Public Health, College Park, MD USA; Westat, Rockville, MD USA; Department of Microbiology and Immunology, University of Maryland School of Medicine, Baltimore, MD USA; American Legacy Foundation, Washington, DC USA

**Keywords:** Vaginal microbiota, Smoking cessation, Cigarette, 16S rRNA gene analysis, Bacterial vaginosis

## Abstract

**Background:**

Smoking has been identified in observational studies as a risk factor for bacterial vaginosis (BV), a condition defined in part by decimation of *Lactobacillus* spp. The anti-estrogenic effect of smoking and trace amounts of benzo[a]pyrene diol epoxide (BPDE) may predispose women to BV. BPDE increases bacteriophage induction in *Lactobacillus* spp. and is found in the vaginal secretions of smokers. We compared the vaginal microbiota between smokers and non-smokers and followed microbiota changes in a smoking cessation pilot study.

**Methods:**

In 2010–2011, 20 smokers and 20 non-smokers were recruited to a cross-sectional study (Phase A) and 9 smokers were enrolled and followed for a 12-week smoking cessation program (Phase B). Phase B included weekly behavioral counseling and nicotine patches to encourage smoking cessation. In both phases, participants self-collected mid-vaginal swabs (daily, Phase B) and completed behavioral surveys. Vaginal bacterial composition was characterized by pyrosequencing of barcoded 16S rRNA genes (V1-V3 regions). Vaginal smears were assigned Nugent Gram stain scores. Smoking status was evaluated (weekly, Phase B) using the semi-quantitative NicAlert® saliva cotinine test and carbon monoxide (CO) exhalation.

**Results:**

In phase A, there was a significant trend for increasing saliva cotinine and CO exhalation with elevated Nugent scores (*P* value <0.005). Vaginal microbiota clustered into three community state types (CSTs); two dominated by *Lactobacillus* (*L. iners, L. crispatus*), and one lacking significant numbers of *Lactobacillus* spp. and characterized by anaerobes (termed CST-IV). Women who were observed in the low-*Lactobacillus* CST-IV state were 25-fold more likely to be smokers than those dominated by *L. crispatus* (aOR: 25.61, 95 % CI: 1.03-636.61). Four women completed Phase B. One of three who entered smoking cessation with high Nugent scores demonstrated a switch from CST-IV to a *L.iners*-dominated profile with a concomitant drop in Nugent scores which coincided with completion of nicotine patches. The other two women fluctuated between CST-IV and *L. iners*-dominated CSTs. The fourth woman had low Nugent scores with *L. crispatus*-dominated CSTs throughout.

**Conclusion:**

Smokers had a lower proportion of vaginal *Lactobacillus* spp. compared to non-smokers. Smoking cessation should be investigated as an adjunct to reducing recurrent BV. Larger studies are needed to confirm these findings.

**Electronic supplementary material:**

The online version of this article (doi:10.1186/1471-2334-14-471) contains supplementary material, which is available to authorized users.

## Background

Cigarette smoking is strongly associated, and often found in a dose-dependent relationship, with risk of bacterial vaginosis (BV) [1–12]. The relationship persists after controlling for other known confounders such as sexual behaviors and alcohol use [13]. BV is a common clinical syndrome in which the protective lactic acid-producing bacteria, mainly species of the *Lactobacillus* genus, are in low relative abundance and are supplanted by a diverse array of anaerobic bacteria [14]. BV sufferers frequently report morbidity, including vaginal odor and irritation, [15] emotional, sexual, and social impacts, [16] and are at higher risk for sexually transmitted infections upon exposure [17]. Conventional therapy consists of nitoimidazoles or clindamycin administered orally or topically [15]. Unfortunately, BV can be highly recurrent [18] with over 50% of women experiencing a symptomatic relapse within 3–12 months following antibiotic therapy [19]. An unexplored intervention for BV is smoking cessation.

Bagaitkar *et al*. cite three mechanisms by which tobacco affects bacterial infections across the human body: physiological and structural changes, increase in bacterial virulence, and dysregulation of immune function [20]. Nicotine and its metabolite cotinine have been detected in the cervical mucus of smokers [21–23]. It is also hypothesized that smoking leads to an accumulation of vaginal amines, [21] which combined with the antiestrogenic effect of smoking [24] predisposes a woman to BV. Women who smoke have significantly lower levels of mid-cycle and luteal phase estradiol compared with non-smokers, [24] and it is well documented that the vaginal microenvironment is influenced by endogenous estrogen [25–27]. In addition, trace amounts of benzo[a]pyrene diol epoxide (BPDE) are found in the vaginal secretions of women who smoke and BPDE significantly increases bacteriophage induction in lactobacilli [28]. Smoking may then reduce the abundance of protective vaginal lactobacilli in part by promoting phage induction.

We hypothesize that the composition of vaginal bacterial communities (termed the vaginal microbiota) is strongly affected by smoking. In this study we compared the vaginal microbiota, as determined by 16S rRNA gene sequencing, between smokers and non-smokers, and we also followed changes in the vaginal microbiota over time in a pilot study of smoking cessation.

## Methods

This manuscript details two separate Phases in our smoking study. The first (Phase A) was a cross-sectional study in which smokers and non-smokers were compared at a single time point. The second (Phase B) was a longitudinal study in which a subgroup of smokers from Phase A were recruited to a smoking cessation pilot study.

### Phase A: cross-sectional study

In 2010–2011, 20 smokers and 20 non-smokers were recruited to a cross-sectional study (single visit) at the Center for Health Behavior Research (CHBR) at the University of Maryland School of Public Health (UMSPH). Inclusion criteria were non-pregnant, non-lactating women, aged 18 to 45 years. Women had to be healthy as determined by medical history, with absence of acute or chronic illnesses, including serious psychiatric disorders or current depression. In addition, participants were excluded if they had used an antibiotic or antimycotic in the prior 30 days or reported a known history of other drug or alcohol dependence in the prior 12 months. Current smoking status was determined by self-report and confirmed by carbon monoxide (CO) exhalation and saliva cotinine measures (described below). Non-smokers also denied smoking in the prior year. Participants completed questionnaires on demographics, nicotine withdrawal, smoking urges, smoking history and tobacco use behavior, depression, nicotine dependence, and reproductive health history.

In a private clinic room, participants self-collected two mid-vaginal swabs (Copan flocked nylon elution-swab and Starplex double headed rayon swab), measured their vaginal pH using the VpH glove (Inverness Medical) and prepared a vaginal smear on a slide for Nugent Gram-stain analysis [29]. The Nugent score reflects the relative abundance of large Gram-positive rods (lactobacilli), Gram-negative and Gram-variable rods and cocci (i.e., *G. vaginalis*, *Prevotella*, *Porphyromonas*, and peptostreptococci), and curved Gram-negative rods (i.e., *Mobiluncus*). This technique allows assessment of relative numbers of bacteria, allowing for classification of bacterial load. It is based on a linear scale ranging from 0–10. A score of 0–3 is considered normal, 4–6 is an intermediate state, and 7–10 is indicative of BV.

Self-collection of mid-vaginal swabs for microbiota analysis has been validated in several studies comparing self-collected to clinician-collected samples [30–32]. We have also validated the use of a home freezer (−20°C) in a vaginal microbiota study which compared cold chain protocols [33].

### Phase B: a pilot longitudinal study of smoking cessation

Phase B participants (n = 9) were recruited using the same criteria as Phase A. Additional inclusion criteria for the Phase B smoking cessation trial were being a current smoker who smoked at least 10 cigarettes/day, no reported period of smoking abstinence greater than three months in the prior year, no history of hypersensitivity to nicotine or adhesives, and motivation to quit smoking. Participants completed questionnaires on demographics, nicotine withdrawal, smoking urges, smoking history and current behavior, depression, nicotine dependence, and reproductive health history at their baseline visit.

### Daily procedures at the participant’s home

Each day for the 12-week study, participants self-collected two mid-vaginal swabs (Copan flocked nylon elution-swab and Starplex double headed rayon swab), measured their vaginal pH (VpH glove, Inverness Medical) and prepared a vaginal smear on a slide for Nugent Gram stain analysis [29]. The two vaginal swabs were stored at the participants’ home in the freezer and the smear was stored in a slide container at room temperature. Participants transported samples to the CHBR weekly in coolers with cold packs to maintain frozen samples. The participants also completed daily diaries which documented time-varying factors including sexual activities, menstrual cycle, feminine hygiene, smoking and nicotine patch use.

### Weekly procedures at the study site

All participants received one-on-one behavioral counseling each week [34] that consisted of an initial 20-minute session focused on setting the target quit date and preparing to quit (baseline visit) and approximately 10-minute weekly sessions thereafter, focused on quitting or preventing relapse. Participants were given a modified copy of the National Cancer Institute’s booklet entitled “Clearing the Air”, a take-home aid for individuals in smoking cessation programs [35].

During the first ten weeks of the study, participants were asked to use Nicoderm CQ® patches daily as an aid to quitting smoking and were individually trained on proper patch application by the study staff. Participants stepped down the nicotine patch levels according to a known efficacious dosing schedule: a 21 mg patch in weeks 1 to 6, 14 mg patch in weeks 7 to 8, and 7 mg patch in weeks 9 to 10 [36]. There was no prescribed patch use in weeks 11–12 which represented a two-week nicotine-free observation period. At each weekly visit, smoking status and patch use was verified using saliva cotinine and CO measurements.

All participants in Phase A and Phase B provided written informed consent. Ethical approval was obtained from the Institutional Review Boards of the University of Maryland Baltimore (UMB) and the UMSPH.

### Biomarkers of smoking

Smoking status was evaluated using both the semi-quantitative NicAlert® saliva cotinine test and Bedfont Micro Smokerlyzer® to measure CO exhalation [37–40]. Breath CO has a 5-6- hour half-life so it is accurate in detecting a 24-hour time frame [41]. The half-life for cotinine is approximately 20 hours and therefore can identify longer term abstinence [42]. The cotinine test can detect six levels of concentrations from 0 to 2000+ ng/ml. In this study, a CO of greater than 8 parts per million (ppm) and a cotinine measurement of greater than 200 ng/ml defined a smoker. When a participant has quit smoking and is actively using the nicotine patch, they are expected to have slightly elevated cotinine levels [43].

### Composition of vaginal bacterial communities

DNA extraction from vaginal swabs, PCR amplification and 454 pyrosequencing of the V1-V3 hypervariable regions of the 16S rRNA genes were performed as previously described [44] using primers 27 F* [45] and 534R [46].

The QIIME split_libraries.py script (version 1.7.0) [44] was used to demultiplex and quality filter sequence reads that had a perfect match to the unique sample-specific barcode sequence by: 1) removing primer and barcode sequences; 2) truncating reads to the first ambiguous base; 3) truncating reads to the first base of a 25 bp sliding window where the average quality within the window dropped below Q25; and 4) including only reads with lengths between 300–600 bp. Quality filtered reads were de-replicated (99% similarity) using the UCLUST software package [47] and potential chimeric sequences were removed using the UCHIME component of UCLUST [48] prior to taxonomic assignment. Genus level taxonomic assignments were performed by using the RDP Naïve Bayesian Classifier [49], and further species level assignments of *Lactobacillus* sp. were performed using higher order Markov Chain *Lactobacillus* species models using the software speciateIT (speciateIT.sourceforge.net) [44]. For each sample, vectors of phylotype proportions were clustered into community state types (CSTs) as previously described by Gajer *et al*. [50].

### Statistical analysis

Fisher’s exact test and logistic regression were used to determine the association between vaginal bacterial CST and smoking status. Factors collected from questionnaires that had been identified on the basis of previous literature and biologic plausibility were evaluated in analyses. To assess differences in the bacterial community structure between smokers and non-smokers, we also utilized the classification and regression tree (CART) analysis. Data were analyzed using STATA/SE 10.0 for Windows (Stata Corporation, College Station, Texas) and the CART was performed in R (R Foundation for Statistical Computing, Vienna, Austria).

## Results

### Phase A: cross-sectional study

Forty women were enrolled in the cross-sectional study with an average age of 29 years (SD: 9.03, range 19–45). Of the 20 smokers, 11 women (55%) reported smoking 1–10 cigarettes per day in the prior 30 days while 9 women (45%) reported 11–20 cigarettes per day. Cotinine and CO levels matched self-reported smoking status (Table 1). There were no differences in self-reported vaginal symptoms between smokers and non-smokers. One smoker reported a diagnosis of BV in the two months prior to her visit. Compared to non-smokers, smokers tended to be older, report a greater number of lifetime sexual partners, and a greater frequency of vaginal douching.

**Table 1:** **Factors associated with smoking status in a cross-sectional study, College Park, MD, n = 40**

	Non-smokers*		Smokers		*P* value**
	n	%	n	%	
Vaginal community state type (CST)^§^					0.04
CST I, *L. crispatus*-dominated	13	65.0	6	30.0	
CST III, *L. iners*-dominated	4	20.0	4	20.0	
CST IV, *Lactobacillus*-deficient	3	15.0	10	50.0	
Nugent Gram stain score					0.02
0-3	17	85.0	10	50.0	
4-6	2	10.0	2	10.0	
7-10	1	5.0	8	40.0	
Vaginal pH^†^					0.13
<=4	10	50.0	4	20.0	
4.1-5.5	1	5.0	2	10.0	
4.6-5.0	4	20.0	3	15.0	
> = 5.1	5	25.0	11	55.0	
Age					<0.01
18-28	17	85.0	5	25.0	
29-39	1	5.0	7	35.0	
40-45	2	10.0	8	40.0	
Ethnicity					0.16
African-American	5	25.0	10	50.0	
Asian/Pacific islander	3	15.0	1	5.0	
Hispanic	3	15.0	0	0.0	
Multi-racial	1	5.0	3	15.0	
White	8	40.0	6	30.0	
Marital status					0.09
Never married	17	85.0	10	50.0	
Married	2	10.0	4	20.0	
Separated, divorced, widowed	1	5.0	5	25.0	
Other	0	0.0	1	5.0	
Education, years					0.06
12	0	0.0	5	25.0	
13-16	18	90.0	13	65.0	
> = 17	2	10.0	2	10.0	
Biomarkers of smoking					
Cotinine, ng/ml					<0.01
0-10	1	5.0	0	0.0	
11-30	17	85.0	0	0.0	
31-100	2	10.0	0	0.0	
101-200	-	-	-	-	
201-500	-	-	-	-	
501-1000	0	0.0	2	10.0	
>1000	0	0.0	18	90.0	
Carbon monoxide exhalation, ppm					<0.01
0-7	20	100.0	0	0.0	
8-10	0	0.0	11	55.0	
11+	0	0.0	9	45.0	
**Self-reported factors**					
Number of cigarettes per day					<0.01
None	20	100.0	0	0.0	
1-10	0	0.0	11	55.0	
11-20	0	0.0	9	45.0	
Symptoms in 24 hours prior to visit					
Odor	5	25.0	3	15.0	0.70
Irritation	0	0.0	0	0.0	-
Itching	0	0.0	0	0.0	-
Burning	0	0.0	0	0.0	-
Pain on urination	0	0.0	0	0.0	-
Discharge	5	25.0	4	20.0	>0.99
Diagnoses in the prior 2 months					
None	19	95.0	17	94.4	0.73
Bacterial vaginosis	0	0.0	1	5.6	
*Trichomonas vaginalis* and *Chlamydia trachomatis*	1	5.0	0	0.0	
Number of male sex partners in the prior 2 months					0.63
0	5	26.3	4	21.1	
1	11	57.9	14	73.7	
2-3	3	15.8	1	5.3	
Number of female sex partners in the prior 2 months					0.73
0	16	94.1	14	93.3	
1	1	5.9	1	6.7	
Lifetime number of sex partners					<0.01
0-4	8	40	2	11.1	
5-7	9	45	1	5.6	
8+	3	15	15	83.3	
Hormonal contraception (HC)					0.19
Other, non-HC	6	33.3	8	57.1	
Oral contraceptive pill	11	61.1	4	28.6	
Injectable	1	5.6	2	14.3	
Frequency of vaginal douching in prior 2 months					0.06
None	18	90.0	12	63.2	
Every now and then	0	0.0	1	5.3	
1–2 times per month	1	5.0	5	26.3	
1 time per week	1	5.0	0	0.0	
More than one time per week	0	0.0	1	5.3	
Sexual activity in prior 24 hours					0.34
No vaginal intercourse	15	75.0	10	50.0	
Vaginal intercourse without a condom	3	15.0	6	30.0	
Vaginal intercourse with a condom	2	10.0	4	20.0	
Menstrual hygiene in prior 24 hours					>0.99
Sanitary napkin, no tampon	11	57.9	11	64.7	
Sanitary napkin only	2	10.5	1	5.9	
Tampon only	4	21.1	4	23.5	
Tampon and sanitary napkin	2	10.5	1	5.9	

^§^CST defined by Ravel et al.^44^

*Non-smoker defined by self-report and 0–200 ng/ml on saliva cotinine test (NicAlert®) and 0–7 ppm on carbon monoxide exhalation (ppm), Bedfont Micro Smokerlyzer®.

**P- values determined by Fisher's exact test.

^†^CarePlan® VpH Test Glove.

**Figure 1:**
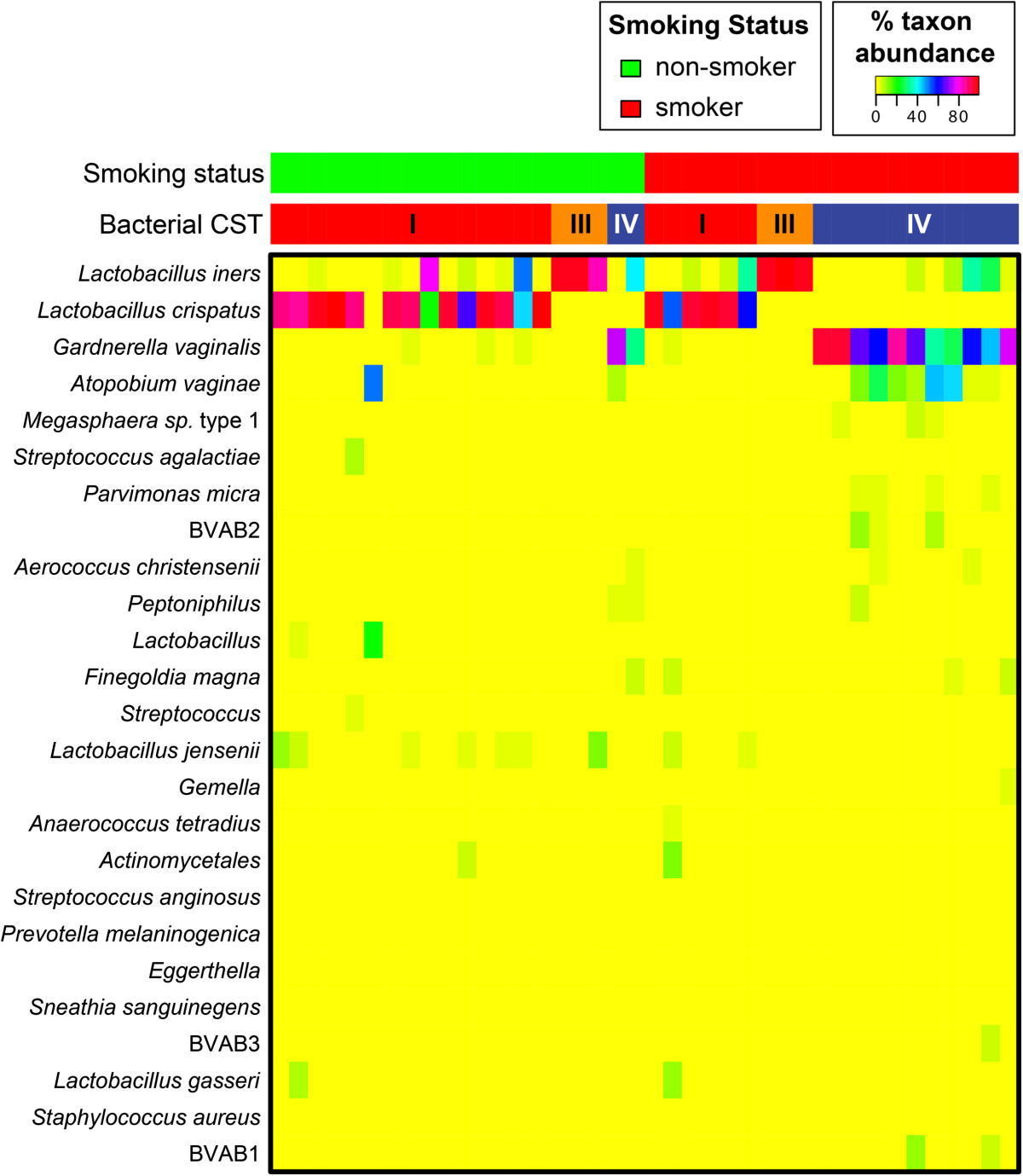
**Heatmap of bacterial relative abundance from 20 smokers and 20 non-smokers sampled cross-sectionally.** Each vertical line represents the bacterial composition of one participant. Smoking status is displayed at top in red and green. Bacterial community state type (CST) are determined vectors of phylotype proportions as previously described by Gajer et al. [50].

Vaginal microbiota clustered into three community state types (CSTs) [44]; two dominated by *Lactobacillus* spp. (*L. iners* (termed CST III)*, L. crispatus* (CST I)), and one lacking significant numbers of *Lactobacillus* spp. and characterized by anaerobic and strictly anaerobic bacteria (CST-IV) (Figure 1). In prior work we split CST IV into CST IV-A and IV-B, [50] however, all but two of the samples were assigned to CST IV-B, and therefore CST IV-A samples were combined to one CST IV group as in Ravel *et al*. [44].

CST was significantly associated with smoking status (*P* value =0.04). Fifty percent of smokers versus 15 % of non-smokers were classified to the low-*Lactobacillus* CST IV (Table 1). Similarly, fewer smokers had a *L. crispatus*-dominated CST I (30% in smokers versus 65% in non-smokers). Smokers tended to have higher Nugent Gram-stain scores (*P* value = 0.02) and higher vaginal pH (*P* value = 0.13) indicative of BV diagnosis compared to non-smokers. Among women observed to have high cotinine concentration (>1000 ng/ml) and CO exhalation (>11 ppm), 56% were categorized to the low-*Lactobacillus* CST IV (*P* value < 0.04). There were also statistically significant trends for both increasing cotinine concentration and CO exhalation with increasing Nugent score (*P* value = 0.004 and *P* value = 0.005, respectively). Smokers may also have had more sexual exposures with 83% of smokers versus 15% of non-smokers reporting a history of eight or more sexual partners (p < 0.01).

In multivariate modeling adjusting for confounders associated with both BV and smoking (vaginal douching and lifetime number of sex partners), women who were observed in the low-*Lactobacillus* CST IV were 25-fold more likely to be smokers than those dominated by the *L. crispatus*-dominated CST I (adjusted odds ratio (aOR): 25.61, 95% CI: 1.03-636.61); Table 2). Age was non-significant in the multivariable model although there were wide divergences in age between smokers and non-smokers. Ninety percent of the non-smokers were age 18–28 versus 75% of the smokers were age 29–45. The model was unable to adjust for contraceptive type because eight of the 40 women declined response on the survey and therefore further limited the sample size of the multivariate model.

**Table 2:** **Odds ratios for factors associated with smoking status, College Park, MD, n = 40**

	OR	95% CI		*P*value	Adjusted OR	95% CI		*P*value
Community state type (CST), dominant bacteria^§^								
CST-I, *L. crispatus*-dominated	REF	-	-	-	REF	-	-	-
CST-III, *L. iners*-dominated	2.17	0.40	11.74	0.37	2.66	0.19	38.04	0.47
CST-IV, *Lactobacillus*-deficient	7.22	1.44	36.22	0.02	25.61	1.03	636.61	0.05
Age								
18-28	REF	-	-	-				
29-39	23.80	2.34	242.29	0.01	-	-	-	-
40-45	13.60	2.15	85.86	0.01	-	-	-	-
Education, years	0.82	0.61	1.10	0.18	-	-	-	-
Douching, past 2 months	5.25	0.93	29.70	0.06	0.81	0.05	13.61	0.88
Currently menstruating	1.00	0.29	3.45	1.00				
Lifetime number of sex partners								
0-4	REF	-	-	-				
5-7	0.44	0.03	5.88	0.54	0.31	0.01	9.84	0.50
8 or more	20.00	2.75	145.48	0.00	40.70	2.46	674.00	0.01
Sexual activity in prior 24 hours								
No vaginal intercourse	REF	-	-	-	-	-	-	-
Vaginal intercourse with no condom	3.00	0.61	14.86	0.18	-	-	-	-
Vaginal intercourse with a condom	3.00	0.46	19.59	0.25	-	-	-	-

^§^CST defined by Ravel *et al.* ^44^

In the CART analysis, bacteria from the genera *Peptostreptococcus* and *Veillonella* were identified as the most important bacterial predictors for smoking status among the 133 bacteria observed.

### Phase B: a pilot longitudinal study of smoking cessation

**Figure 2:**
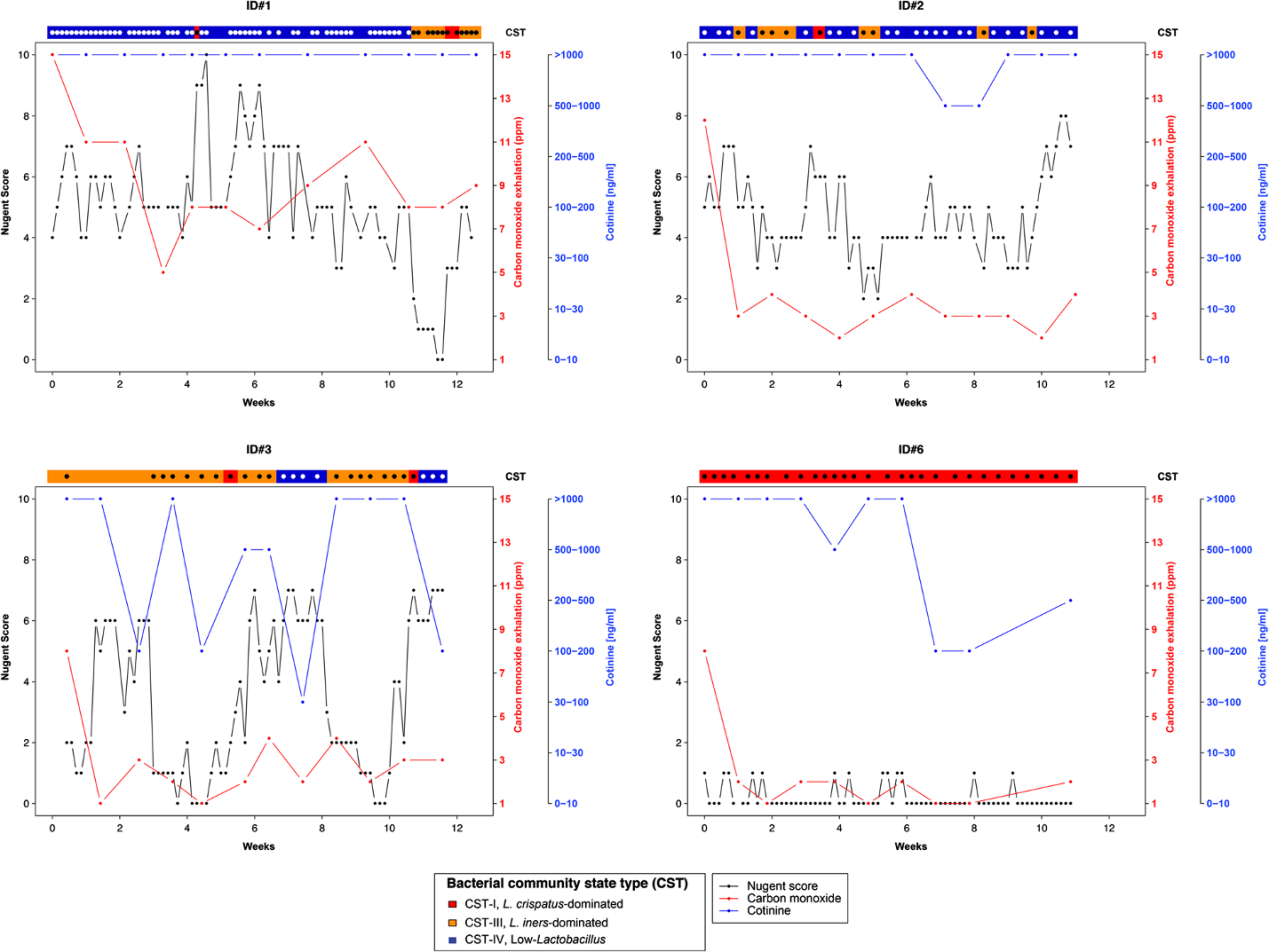
**Longitudinal trajectories of the four women who completed the 12-week smoking cessation intervention.** See key for Nugent Gram stain score, carbon monoxide, cotinine and bacterial community state type assignments.

Of the nine women who enrolled in Phase B, four completed the 12-week study, three discontinued at 5–8 weeks, and two dropped out immediately. None of the women in Phase B reported a clinician’s diagnosis of symptomatic BV or antibiotic use during the study. Cotinine and CO levels of enrolled women suggest they maintained nicotine patch use (high cotinine levels) and quit smoking or reduced smoking (low CO levels) (Figure 2). Even among those women who were lost to follow-up at weeks 5–8, cotinine and CO levels suggest good compliance with smoking cessation (data not shown).

Of the four women who completed the 12-week study, three entered the study with high Nugent scores and vaginal microbiota belonging to the low-*Lactobacillus* CST IV (Figure 2). Of these three women, one woman (ID#1) demonstrated a possible response to smoking cessation. At week 10, coinciding with completion of the nicotine patch and smoking cessation program, her CSTs switched from CST IV to CST III (a *L. iners*-dominated) and CST I (*L. crispatus*-dominated) with a concomitant drop in Nugent scores. She did not report a vaginitis diagnosis during the study or in the two months prior to study entry, nor any antibiotic use during the study or any change in behaviors which she documented on the daily diaries. She reported intermittent vaginal odor and discharge on daily diaries but had no such complaints in the final two weeks of observation when she had ceased smoking, finished the nicotine patch protocol and switched to the *Lactobacillus*-dominated CSTs. The other 2 women (ID#s 2 and 3) fluctuated between CST IV and CST III. The fourth woman (ID#6) had low Nugent scores with *L. crispatus*-dominated CST I throughout (Figure 2). Among the three women that did not complete follow-up, Nugent scores fluctuated between low and intermediate categories. Longitudinal multivariate modeling was not possible in Phase B due to the small sample size (n = 4) of this pilot study, and therefore, we are limited to descriptive findings.

## Discussion

The cross-sectional study (Phase A) suggests that women who smoke cigarettes are significantly more likely to have a vaginal microbiota characterized by low proportions of *Lactobacillus* spp. In Phase B, with one of three women shifting from a vaginal *Lactobacillus*-deficient CST to a *Lactobacillus*-dominated CST during smoking cessation without the aid of antibiotics, we hypothesize that smoking cessation could benefit some women struggling with recurrent BV. In order to establish the causal association that smoking directly affects the vaginal microbiome and recurrence of BV, a larger smoking cessation study design is needed that also includes clinical evaluation for BV.

Several participants in Phase B fluctuated between a low-*Lactobacillus* state and a *L. iners* dominated CST with ID#1 appearing to transition fully to a *L. iners*-dominated CST. There has been recent species-specific attention to *L. iners*[51, 52]*L. iners* is commonly found in the vagina [44] and has been associated with both BV and healthy states [53–56]. In addition, *L. iners* is often the first *Lactobacillus* species to recover after treatment for BV [46, 56]. Our group’s prior work suggest there are strains of *L. iners* which are highly stable over time while others are associated with a rapidly changing vaginal microbiota tending toward a BV state [46, 50]. Ongoing work is evaluating the genomic heterogeneity of *L. iners* and if different strains are associated with STI or BV outcomes [57].

This pilot study provides important preliminary data for future studies. Attrition was high among participants attempting smoking cessation in Phase B (55%), although those who remained in the study were very compliant with smoking cessation, nicotine patch use, attending weekly counseling sessions, and daily collection of vaginal swabs, vaginal pH, vaginal smears and diary entries. Women who were lost to follow-up were also compliant with smoking cessation as indicated by biomarkers. Loss to follow-up rates greater than 30% are not unusual in smoking cessation trials [58–61], and therefore a future study may need to recruit two-fold more women as our study indicates. Weekly visits confirmed that samples were collected in the week stipulated. We advocate that future studies include frequent self-collection of vaginal swabs (daily or 2–3 times weekly) with careful collection of the menstrual cycle, antibiotic use and behavioral data [50, 62, 63]. In addition, longer duration of follow-up (>12 weeks) is likely necessary to capture all women if their microbiome is to rebound to *Lactobacillus*-dominated CSTs.

There are a number of limitations to this study. The research was designed as a pilot, and therefore, sample size and funds were limited. We were unable to conduct broad testing for sexually transmitted infections, and larger studies may be able to detect other known CSTs, such as CSTs dominated by *L. gasseri* and *L. jensenii*. It should also be noted that there are known racial and ethnic differences in nicotine metabolism, which may affect biomarkers of smoking exposure [64–66]. Cotinine and carbon monoxide levels matched self-report of smoking in our study. Also due to sample size, we were unable to control for important confounders. For example, another important factor which may be driving the inverse association between *Lactobacillus*-dominated CSTs (and also Nugent scores) with smoking status is hormonal contraception (HC). Use of HC in most epidemiological studies has been associated with a reduced risk of bacterial vaginosis [67]. Sixty percent of non-smokers versus 25% of smokers were using HC. The differences in HC use likely reflects clinicians’ prescribing patterns in which smokers are not prescribed HC due to potential cardiovascular side effects [68]. Further, smokers tended to be older (over age 40 and possibly peri-menopausal) and therefore less likely to use HCs. It was not possible for this analysis to use HC in statistical modeling because eight of the 40 women in Phase A declined to answer the contraceptive questions and the sample size became prohibitive. In addition, HC use was not different between smokers and non-smokers in univariate analysis (*P* value =0.19). It remains important for future studies to collect HC formulation data and control for it in analysis.

It was also surprising to observe 5% of the non-smokers versus 40% of smokers had high Gram stain scores indicative of BV. This could have been the result of recruitment of “healthier” women than an average non-smoking cohort, and therefore, the non-smoking group was biased by women with *Lactobacillus*-dominated microbiota. However, non-smokers and smokers were recruited using the same methods of advertising and outreach, and more importantly, our data demonstrated that there were statistically significant dose-responses for increasing cotinine concentration and CO exhalation with increasing Nugent score. Numerous studies indicate BV is more common in smokers than non-smokers, smoking has a dose-dependent relationship with BV and the associations persist after controlling for confounders [1–12]. The current study also utilized saliva cotinine and carbon monoxide exhalation measures (which prior studies have not collected) coupled with extensive surveys to properly categorize women as smokers and non-smokers. A larger study with more representative sampling, coupled with biomarker detection of smoking, could resolve this issue.

A major strength of this study is that we are able to provide preliminary data for future studies and self-report of smoking status was confirmed by biomarkers. The study also utilized comprehensive questionnaires, weekly counseling in smoking cessation and high-throughput DNA sequencing technologies. Participants self-collected vaginal swabs daily so the dynamics of the microbiota could be intensively followed in smoking cessation.

## Conclusion

To our knowledge, this is the first study to assess differences in the vaginal microbiota between smokers and non-smokers and to begin to assess how smoking cessation affects the vaginal microbiome. We found smoking is associated with a vaginal microbiota which has low proportions of *Lactobacillus* spp, however future research is needed to establish if smoking is causally related to BV and to assess if smoking interventions could positively affect the vaginal microbiome. If smoking cessation proves to reduce incidence, persistence or recurrence of BV, it may also offer an additional incentive for women to quit smoking.

## Authors’ original submitted files for images

Below are the links to the authors’ original submitted files for images. Authors’ original file for figure 1 Authors’ original file for figure 2
