# Supplementary figures and images for: Association between cigarette smoking and the vaginal microbiota: a pilot study
Source: BMC Infect Dis. 2014 Aug 28;14:471. doi: 10.1186/1471-2334-14-471 (PMC4161850; doi:10.1186/1471-2334-14-471)

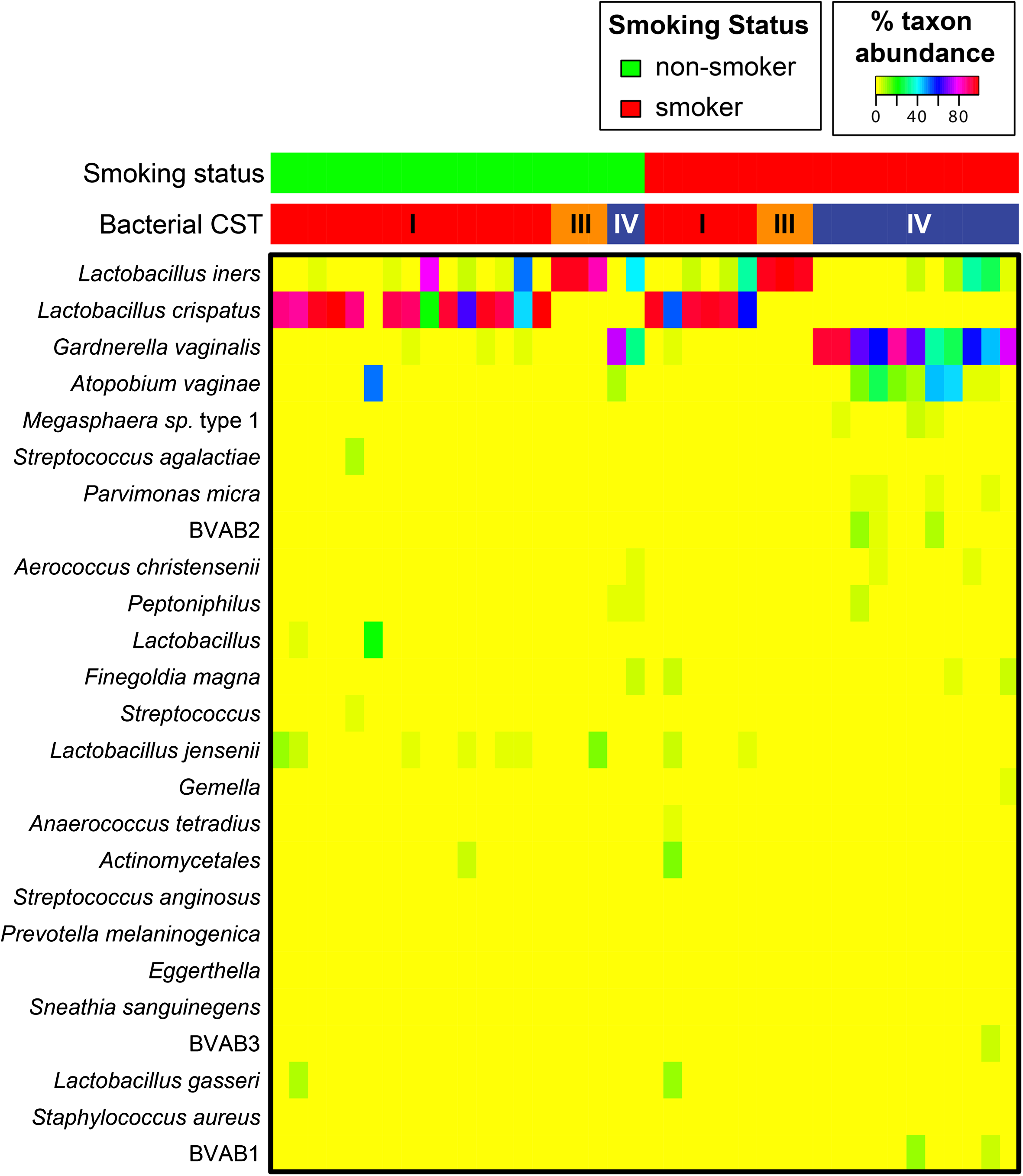

Supplement: Supplementary file 1 — Authors’ original file for figure 1 [file 12879_2014_3781_MOESM1_ESM.tif]

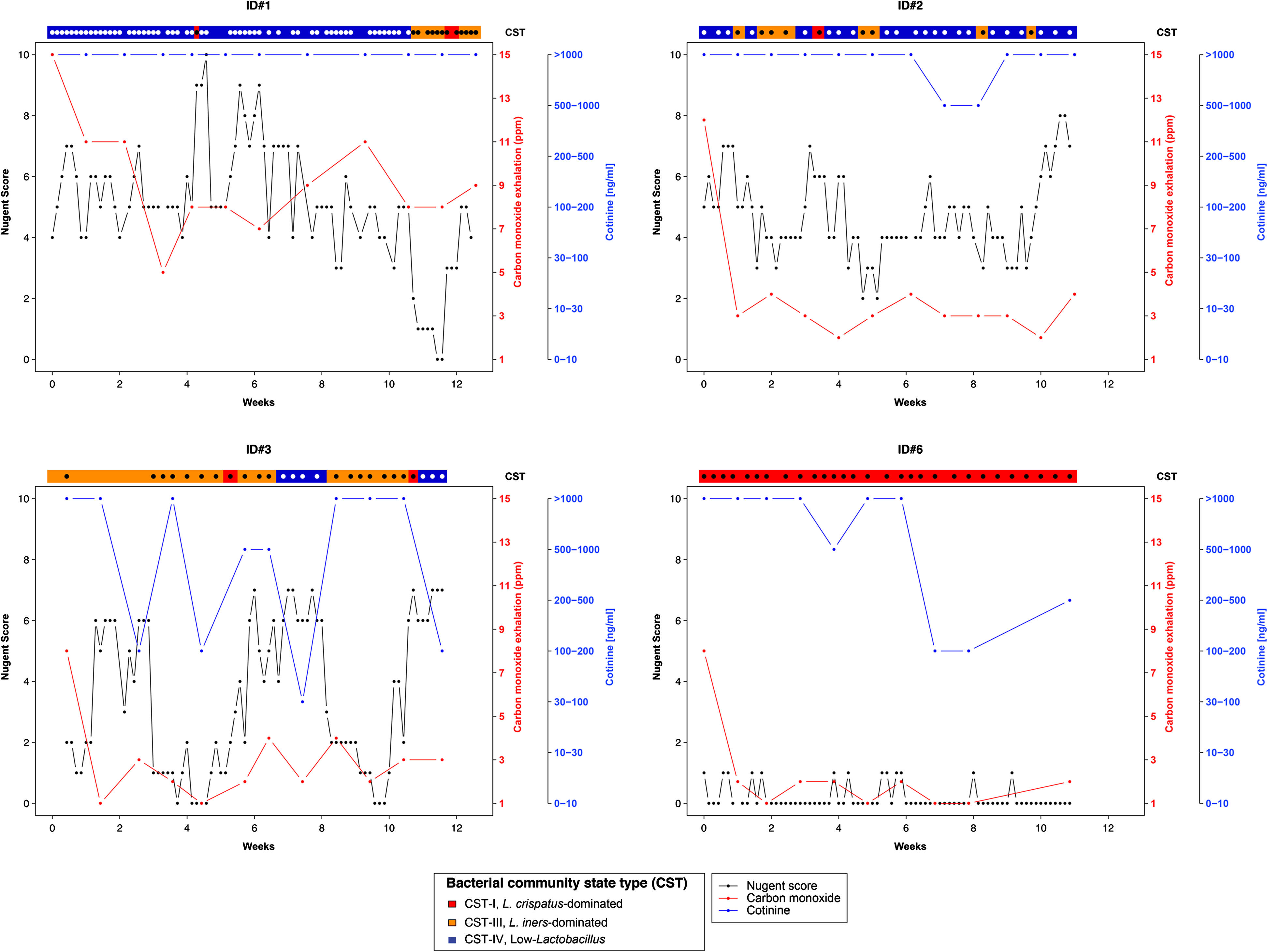

Supplement: Supplementary file 2 — Authors’ original file for figure 2 [file 12879_2014_3781_MOESM2_ESM.tif]
